# “Well, that Was Pretty Clever!”—Ethnic Minority Women’s Views on HPV Self-Sampling Devices for Cervical Cancer Screening: Attitudes to Brushes, First-Void Urine, and Menstrual Blood Devices

**DOI:** 10.1007/s40615-024-01963-9

**Published:** 2024-03-12

**Authors:** Signe Ruby Hald, Camilla Rahr Tatari, Pia Kirkegaard, Mette Tranberg, Berit Andersen, Camilla Palmhøj Nielsen

**Affiliations:** 1 Department of Public Health Programmes, Randers Regional Hospital, University Research Clinic for Cancer Screening, Randers, Denmark; 2 Department of Public Health, Aarhus University, Aarhus, Denmark; 3 Department of Clinical Medicine, Aarhus University, Aarhus, Denmark; 4 Department of Pathology, Randers Regional Hospital, Randers, Denmark; 5 DEFACTUM, Public Health Research, Central Denmark Region, Aarhus, Denmark

**Keywords:** Self-sampling, Human Papillomavirus testing, Cervical cancer screening, Social inequality, Emigrants and immigrants, Qualitative research

## Abstract

**Background:**

Ethnic minority women are less likely to participate in cervical cancer uteri (CCU) screening compared to native women. Human Papillomavirus (HPV) self-sampling kits for CCU screening may be a potential strategy to increase participation. This study aimed to explore views and attitudes on four different types of self-sampling kits (two brushes, a first-void urine device, and a menstrual blood device) among non-Western ethnic minority women living in Denmark.

**Methods:**

The study was a social science single case study based on focus group interviews with 30 women aged 32–54 with non-Western background from a deprived area. A phenomenological approach was applied to describe the phenomenon “self-sampling” as seen from the women’s lifeworlds. The interviews were transcribed verbatim and analysed using systematic text condensation.

**Results:**

The women expressed significant interest in the possibility of using HPV self-sampling kits as an alternative to being screened by their general practitioner. They were particularly motivated to use the non-invasive self-sampling kits for CCU screening as they were deemed suitable for addressing cultural beliefs related to their bodies and virginity. The women expressed interest in the use of the invasive self-sampling kits but were cautious, primarily due to lack of confidence in correctly performing self-sampling with a brush and due to cultural beliefs.

**Conclusion:**

The use of non-invasive self-sampling kits, such as a first-void urine collection device and menstrual blood pad, represents a promising solution to overcome cultural barriers and promote greater equality in CCU screening participation among non-Western ethnic minority women.

## Introduction

In many Western European countries, organised cervical cancer uteri (CCU) screening programmes can significantly reduce incidence and mortality rates of the disease [1]. Nevertheless, even in countries offering free and equal access to screening for all women, social inequalities in screening participation persist being associated with ethnic minority background and low socioeconomic status [2, 3]. Compared with the native population, some studies [4, 5] revealed an increased risk of CCU among immigrants, especially among those from non-Western countries. These women may have a higher background risk of CCU due to high Human Papillomavirus (HPV) prevalence [4, 5] combined with poor or no access to screening in their home countries [6], while low participation in CCU screening in the new country may be a supplemental explanation [7].

Barriers for attending CCU screening can be related to insufficient knowledge, language barriers, fatalistic beliefs about cancer, discomfort with the gynaecological examination, and taboos related to female genitals and sexuality [8, 9]. Some of these barriers can be overcome by offering women the opportunity to use a self-sampling kit to collect a vaginal, urine, or menstrual blood sample in their own home before returning it for high-risk HPV testing at the laboratory (HPV self-sampling). Considerable evidence supports that under-screened woman, who do not engage in clinician-based screening, can be reached by offering them the opportunity to self-collect a vaginal sample [10]. Consequently, vaginal self-sampling is already implemented in routine screening programmes in some countries including Denmark and is a critical component of the WHO’s global strategy to eliminate CCU [11]. Tranberg et al. [12] proved increased screening participation across socioeconomic groups by offering vaginal sampling using the dry Evalyn Brush device. However, non-Western ethnic minorities were less likely to use the vaginal brush than Western immigrants and native Danish women [12]. In recent years, HPV self-sampling has seen substantial advancements, with emerging methods like a first-void urine device [13] and a specially designed menstrual blood pad [14] potentially serving as alternatives to reach underserved populations. However, studies about the views and attitudes of using a first-void urine device and a specially designed menstrual blood device among non-Western ethnic minorities are sparse. Therefore, this study aimed to explore views and attitudes on different self-sampling devices being two dry vaginal brush devices, a first-void urine collection device, and a modified menstrual blood pad among non-Western ethnic minority women living in a deprived area in Denmark.

## Methods and Materials

### Setting

This study was conducted in Gellerup, a socially deprived suburban area located in Aarhus. Aarhus is the second-largest city in Denmark with a population of more than 360,000 residents [15]. Gellerup itself has over 6000 residents and is characterised by an unemployment rate of 54%, with 81.3% of its residents coming from non-Western countries [16].

The Danish health care system is founded on the principle of providing free and equal access to preventive care and treatment for all residents [17]. This includes the CCU screening and any subsequent follow-up visits and treatments. In Denmark, women are invited to participate in CCU screening every third year between the ages of 23 and 49, and every fifth year when aged 50–64 [18]. The invitation is sent via digital mail, recommending women to schedule an appointment with a general practitioner (GP) for a cervical cytology specimen collection during a gynaecological examination [18]. Non-responders receive two reminders. Since 2022, the Central Denmark Region has together with the second reminder offered non-participating women the opportunity to order a vaginal self-sampling kit (Evalyn Brush) [18, 19]. All communication and written materials regarding CCU screening are provided in Danish with a link to an English version.

### Presentation of Self-Sampling Screening Devices

In the study, we introduced four different HPV self-sampling kits to the participants. Detailed descriptions of these kits can be found in Table 1. For images of the devices, please consult the provided references.

**Table 1:** Specifications of the introduced HPV self-sampling kits

Device	Specification
Viba: Dry brush	The dry Viba-Brush is 20 cm long and consists of a pink handle with a white brush for collecting cell material. The Viba-Brush should be inserted into the top of the vaginal canal to a depth of at least 5 cm, after which it should be rotated five times. The Viba-Brush does not have any indicators to assist the woman in securing the correct depth. After extraction, the brush head should be released into a plastic cap and returned by postal mail for HPV testing at a laboratory [20]
Evalyn: Dry brush	The dry pink Evalyn Brush is used by inserting it vaginally. Afterward, the brush should be rotated five times and removed gently. To ensure correct sampling and to reassure the woman during the process, the brush has built-in features such as indicator wings used to indicate the depth to which the brush should be inserted vaginally, as well as feedback “clicking sounds” that guide the woman along the way. After extraction, the brush head should be released into a plastic cap and returned by postal mail for HPV testing at a laboratory [19]
Colli-Pee: First-void urine	The Colli-Pee is a first-void urine collection device. The urine sample is obtained by using a sample container and a funnel, where the funnel should be securely attached to the sample container. The woman can then urinate into the funnel. The funnel will automatically collect the correct amount of urine (10 ml), so the woman does not need to interrupt the flow of urine. The funnel should then be returned by postal mail for HPV testing at a laboratory [21]
Q-Pad: Modified Menstrual Pad	A Q-Pad is a modified menstrual pad with a built-in paper blood strip that can collect small amounts of blood. The woman uses the menstrual pad (Q-pad) during her period, after which she can remove the paper strip, send in the strip by postal mail for HPV testing at a laboratory and then dispose of the pad [22]

### Design and Participants

The study was designed as a social science single case study which is an investigation of a contemporary phenomenon occurring within a particular context [23]. The case was geographically and culturally delimited to investigating views and attitudes on self-collected samples among women of different ethnic backgrounds residing in Gellerup. The phenomenon to be understood was self-collected samples among women of different ethnic backgrounds in Denmark, with the aim of deriving knowledge that can contribute to the development of a national Danish screening programme. The study adopted a phenomenological approach to capture the aspects of the lifeworlds related to screening for CCU [24, 25]. Data was collected through focus groups interviews (FGI). This method enabled the researchers to gain insight into the participants’ understandings, experiences, and barriers to participation, as well as to observe the variations and interplay between perceptions and barriers towards self-sampling and screening [26, 27].

The recruitment of participants was carried out in cooperation with a local non-governmental organisation with primarily women with an ethnic minority background who volunteer in the local community to support isolated and vulnerable women. Specific inclusion criteria were having an ethnic minority background, residing in the area, being between the ages of 23 and 64, and having the ability to speak and understand Danish. The number of informants followed Malterud’s concept of information power [28]; a sampling method that emphasises shifting focus away from the number of participants and towards the quality of the data provided by the participants [28]. Each FGI contained 6–9 participants and was held in the local area.

Thirty women participated in the FGI. Characteristics of the participants in each FGI can be seen in Table 2. The length of each FGI included debriefing and varied from 74 to 80 min. Seven participants failed to disclose their age. A total of twenty-three participants reported the age ranging from 32 to 54 years (average:43.8 years). Eight participants failed to disclose their country of origin and the rest reported five different non-Western countries with a majority of Somali women. Over half of the participants had prior screening experience, primarily through opportunistic screenings linked to intra-uterine device insertion and removal, or postpartum check-ups.

**Table 2:** Characteristics of the focus group interviews

Interview	Length in minutes	Number of participants	Age interval	Country of origin
Focus group interview 1	74	6	35–53	Pakistan (*n* = 1) Somalia (*n* = 5)
Focus group interview 2	77	8	41–54 (missing *n* = 2)	Saudi Arabia (*n* = 1) Somalia (*n* = 6) Missing (*n* = 1)
Focus group interview 3	80	9	38–45 (missing *n* = 5)	Lebanon (*n* = 1) Morocco (*n* = 1) Somalia (*n* = 2) Missing (*n* = 5)
Focus group interview 4	80	7	32–52	Somalia (*n* = 5) Missing (*n* = 2)

### Interviews

Before conducting the FGI, the participants were introduced to the aim of the study and the framework of the FGI by the researchers facilitating the interviews. Additionally, all participants received a verbal presentation of their rights as participants, including information on how to withdraw their contribution and contact information, before consent to participate was given.

A semi-structured interview guide was discussed and developed by the researchers. It served as a guide for addressing specific topics while encouraging participant interaction and free communication without interrupting each other [29]. The interview guide addressed self-sampling, and the participants were asked about their perspectives on the earlier described four different HPV self-sampling kits: Viba-Brush [20], Evalyn Brush [19], Colli-Pee [21], and the Q-Pad [22]. The interview guide is shown in Table 3.

**Table 3:** The interview guide

Topic	Interview question
Brief overview of the test procedure at the general practitioner	
Test at the general practitioner	What do you think about this? Any reactions, comments, or questions? Can you envision yourself participating in this manner?
Brief overview of two self-sampling kits with brushes. The participants are introduced to the use of the brushes, with examples of the brushes and instructions provided	
Viba and Evalyn – Dry brushes: attitudes and views	What do you think about these? Do you prefer one over the other? Could you see yourself using one of them? Which one? Preference?
Brief overview of a urine device. They have the opportunity to personally explore the test with the accompanying instructions	
Colli-Pee – First-void urine: attitudes and views	What do you think about this? Do you have any experiences with urine samples in other contexts? What are your thoughts on using urine to test for HPV? Could you imagine using it yourself?
Brief overview of the menstrual pad device. They have the opportunity to personally explore the test with the accompanying instructions	
Q-Pad—Modified menstrual pad: attitudes and views	What do you think about this? What are your thoughts on using menstrual blood as a test for HPV? Could you imagine using it yourself?
Overall impression of self-sampling kits	What pros and cons do you see in the different methods you have been presented with? Which of the different methods could you best imagine using yourself?

The FGI were conducted and facilitated by Signe Ruby Hald (SRH) and Camilla Rahr Tatari (CRT) between February and March 2023. Prior to the interviews, the participants, SRH, and CRT had lunch together, which was provided by the researchers. During the lunch, each participant completed a background questionnaire, which covered their experiences with CCU and screening status. As an introduction to the FGI, the participants were encouraged to openly share all aspects of their thoughts and attitudes and discuss their opinions and disagreements with each other. CRT held a brief presentation about HPV, CCU, and screening, using simple hand-drawn illustrations to explain the natural progression of HPV-related CCU and the biomedical principles behind the HPV vaccine and CCU screening. The purpose was to establish a shared understanding of the topics as a basis for further discussions. The FGI ended with a debriefing, providing the participants with a final opportunity to share their thoughts, ask questions, and comment on their interview experience. Additionally, they were acknowledged for their participation and given written contact information on the researchers to be used if needed.

### Analysis

Interviews were audio-recorded and transcribed verbatim by SRH and CRT checked the transcription. In a few cases, it was not possible to make verbatim transcriptions when the informants spoke their mother tongue. Malterud’s systematic text condensation [30] was conducted using the software NVivo 1.5.2 [31, 32]. The analysis contained of four steps: (1) overall impression, (2) identifying and sorting meaning units, (3) condensation, (4) synthesis [30]. SRH and CRT discussed their overall impressions of the data after each interview and upon completing data collection. SRH conducted in-depth analyses of all transcriptions in close collaboration with CRT, who also separately performed an in-depth analysis of the transcription from the second FGI. SRH specialised in qualitative methodology during her master’s degree in Public Health Science, while CRT gained extensive experience with qualitative methodology from previous publications and her PhD degree, where she focused specifically on qualitative research. Additionally, the analyses were presented and discussed with co-authors Camilla Palmhøj and Pia Kirkegaard. This process ensured transparency in the analysis and validation of the findings.

### Ethics

Research involving participants in potentially vulnerable situations requires ethical considerations. It has been crucial to ensure that the participants have not felt shame, worry, inferiority, guilt, or any other unintended emotions during the interviews, which could potentially arise from non-participation in screenings or lack of knowledge. SRH and CRT took this into account by approaching the women with an open-minded, respectful, and curious attitude, thus creating a safe space for the FGI. To achieve this, the FGI were conducted in a community centre with which the informants were familiar, allowing them to feel at ease during the interviews. During the introduction to the FGI, it was made clear that all opinions, attitudes, views, and questions were welcome, and that there were no right or wrong answers. Additionally, it was explicitly communicated, on several occasions, that participation in screening is a voluntary choice, where both the advantages and disadvantages should be considered before making a decision to participate [33, 34].

## Results

The following categories: 1. “The boundaries of the body”, 2. “To do it yourself”, and 3. “Trust in new self-sampling methods” with subcategories emerged from the analysis of the data. An overview of categories, subcategories, and selected quotes are illustrated in Table 4.

**Table 4:** Overview of categories, subcategories, and selected quotes

Category	The boundaries of the body			
Subcategory	**“We do not know our bodies”**	**“As soon as we avoid having to put something up inside us”**		**“But I wouldn’t do it before I had children”**
Selected quotes	“I think you need to know your body a lot when doing it” “I don't think that we know it [the body]” “I dare not touch myself” “Considering our Muslim beliefs, I’d prefer the urine test. I don’t like such intimate contact with my body, and the idea of suddenly having to do all of that touching is uncomfortable. Personally, I wouldn’t feel comfortable using those brushes, but a urine test would be fine”	“I think the biggest difference for me is that I don’t have to insert anything” “I completely agree with the others that the one with the urine sample would be preferred mostly because, well, as you say yourself, it comes naturally, and you don’t have to insert all sorts of things in all sorts of places and so on and so forth”		“But I wouldn’t do it before I had children. I’ve said no every time I was offered (screening). No way. There shouldn’t be anyone ‘down there’ looking at me. No thanks. But today, I can be a bit more open to it” “That’s exactly how I’ve been part of it [screening] before, at the 8-week follow-up after giving birth. I’m not really fond of those check-ups, so I hope other ways of getting it checked will be found” “I’ve got a pair of kids, yet I’ve never been involved. Seems like it’s only after my postpartum check-ups that I’ve shown up, but never when I’ve been invited”
Category	**To do it yourself**			
Subcategory	**“You have to constantly think; have I made a mistake?”**		**“You can’t do anything wrong”**	
Selected quotes	“And if you insert it four centimetres, isn’t that enough?” [Viba—Dry brush] “You never know with those five centimetres, and then you have to twist, and what if you make a mistake. You have to constantly think; have I made a mistake?” [Viba—Dry brush]		“You can’t do anything wrong”, [Evalyn: Dry brush] “… it has this stopper, so you are not in doubt whether you’re in the right place. I believe it’s safer or easier to use than the other one” [Evalyn: Dry brush] “So I think it’s the easiest method, because you’re used to using a menstrual pad” [Q-Pad: Modified menstrual pad]	
Category	**Trust in new self-sampling methods**			
Subcategory	**Attitudes to blood**		**Attitudes to urine**	
Selected quotes	“If you undergo a general examination, always, they take blood, so they know what type of disease it is” “So you have more security and are more certain that you have sent a proper sample”		“…It’s not the system I’ve learned, so I don’t trust the urine too much, because I’m used to, for example, going to the GP if I have a urinary tract infection, and then taking the test for the urinary tract infection” “I actually think that many people would prefer this [urine test] here”	

### The Boundaries of the Body

#### “We Do Not Know Our Bodies”

During the presentation of the brushes, some of the women expressed that using them presupposed having a great knowledge of their own bodies. A woman in the second FGI expressed, “I think you need to know your body a lot when doing it”. while another woman continued in the same FGI: “I don’t think that we know it [the body]”. Several of the women described that they experienced a discomfort to touch themself in order to use a brush self-sampling kit. Overall, they showed a distance to their genitals and reproductive organs and said they were afraid to touch themself in “that area”. Therefore, the women perceived it as a barrier that they had to perform the process themselves when applying an invasive self-sampling device. It was hence considered an advantage if the self-sampling did not require them to touch and feel their own body.

#### “As Soon as We Avoid Having to Put Something Up Inside Us”

A large proportion of the women were keen on exploring the self-sampling devices, which enable testing without requiring insertion, such as the urine device and the menstrual pad. Several women expressed that it made a significant difference to them whether the sample had to be inserted and collected vaginally, as emphasised in this quote from the first FGI: “I think the biggest difference for me is that I don’t have to insert anything”. Thus, the women showed great interest in the urine device and menstrual pad and, as these self-collected samples did not require insertion. An illustration on this point came from the first FGI: “I completely agree with the others that the one with the urine sample would be preferred mostly because, well, as you say yourself, it comes naturally, and you don’t have to insert all sorts of things in all sorts of places and so on and so forth”. The desire not to have to insert the sampling device vaginally was explained by the fact that the women associated this with discomfort, which they would like to avoid. Furthermore, it was mentioned that these methods, which did not require insertion, were particularly suitable for unmarried women who had not had sexual contact with a partner.

#### “But I Wouldn’t Do It Before I Had Children”

Some women expressed that their relationship with their bodies had changed after they had grown older and given birth. The women expressed that motherhood had altered their thoughts and perceptions regarding participating in screening. An example of this perspective was stated in the third FGI: “But I wouldn’t do it before I had children. I’ve said no every time I was offered (screening). No way. There shouldn’t be anyone ‘down there’ looking at me. No thanks. But today, I can be a bit more open to it”. It was a reflection shared by several of the women that participation in screening became a possibility only after they had given birth. Being pregnant and giving birth had given some of the women increased familiarity and experience with gynaecological examination. It was thus evident that preferences regarding self-collected samples were nuanced according to the women’s stages in life. Furthermore, some women expressed that they wished these alternative options had been available before they gave birth themselves, as this would have made them more prone to CCU screening participation.

### To Do It Yourself

#### “You Have to Constantly Think; Have I Made a Mistake?”

Many of the women expressed concerns about performing the self-collected sample with the Viba-Brush correctly. The women emphasised that it was difficult to know whether the insertion was in accordance with the 5 cm specified in the instructions. One woman from the second FGI stated: “And if you insert it four centimetres, isn’t that enough?” while another woman in the fourth FGI expressed: “You never know with those five centimetres, and then you have to twist, and what if you make a mistake. You have to constantly think; have I made a mistake?” For these reasons, the women were sceptical about using the Viba-Brush, as they imagined that using this self-sampling kit would raise doubts about whether the sample had been conducted correctly. The women did not express the same concerns about performing the remaining three samples correctly after they were introduced in the FGI.

#### “You Can’t Do Anything Wrong”

When introducing HPV self-sampling, it became clear that it was important for the women to feel confident in having correctly performed a self-collected sample. Throughout the introduction of the Evalyn Brush, it became evident that it was more intuitive to use compared to the Viba-Brush. Several of the women explained that they particularly found the “clicking” sounds useful, as well as the “wings” that blocks the insertion after 5 cm. One woman stated in the third FGI: “You can’t do anything wrong”, while another explained in the same FGI that: “… it has this stopper, so you are not in doubt whether you’re in the right place. I believe it’s safer or easier to use than the other one”. Many of the women therefore pointed out that the Evalyn Brush seemed easy to use, and that these integrated features could contribute to the feeling of security when the sample was taken. It was also mentioned for the menstrual pad and the urine sample that the devices seemed easy to use. The women specifically mentioned that the menstrual pad seemed easy to use, as they had previously used a pad during menstruation. This was expressed in the fourth FGI: “So I think it’s the easiest method, because you’re used to using a menstrual pad”.

### Trust in New Self-Sampling Methods

#### Attitudes to Blood

A few of the women stated that they considered blood testing a safe way to detect diseases. One woman in the fourth FGI said that doctors usually use blood tests to check for diseases: “If you undergo a general examination, always, they take blood, so they know what type of disease it is”. In continuation of this, other women pointed out that blood can reveal a lot within the body. Additionally, there was a general perception that it was not considered a problem to handle and send one’s own blood. For these reasons, the women showed interest and acceptance of the menstrual pad as a new screening method. Despite their strong preference for the menstrual pad, a few women were unable to choose this option due to factors such as irregular menstruation cycles, absence of menstruation, or reaching menopause.

#### Attitudes to Urine

While the majority of the women were positive about the urine sample as a testing method, a few of them expressed distrust in the urine sample as a screening method. During the presentation of the self-sampling kit, these few women expressed that they could not trust the sample for two specific reasons. Firstly, one woman in the second FGI mentioned that she was accustomed to submitting a urine sample for urinary tract infections. As a result, she was not convinced that a urine sample could also be used for screening for CCU: “…It’s not the system I’ve learned, so I don’t trust the urine too much, because I’m used to, for example, going to the GP if I have a urinary tract infection, and then taking the test for the urinary tract infection”. Another woman explained that in her opinion, it would be better to have a test performed at the GP because she believed it would be a more accurate way as it collects the “mucus”. When taking a urine sample, she couldn’t imagine that there would be enough “material” collected with this method. Despite this scepticism among a minority of the women, the urine sample received a very positive response as an alternative to a sample taken by a GP.

## Discussion

This qualitative study is to our knowledge the first to explore viewpoints regarding self-sampling for CCU screening among non-Western ethnic minority women in Scandinavia. Additionally, it was the first qualitative investigation into attitudes and views related to the menstrual pad as a self-sampling approach in this sub-group of women.

Regardless of device type, the study participants’ attitudes and views expressed significant interest in the possibility of performing a self-sampling kit instead of seeking a GP for a cervical cytology sample. They showed particular interest in the non-invasive menstrual blood pad and urine device, as these sampling procedures did not interfere negatively with cultural attitudes and perspectives related to a woman’s body and virginity.

### Comparison with Other Studies

A main result in this study was the participants’ hesitance to engage in self-touch with invasive self-sampling devices. This result has been noted across several studies involving women from various backgrounds and origins [35–37]. While these studies have not specifically targeted ethnic minorities, this trend suggests that a cultural notion about the body could be a universal barrier towards invasive self-sampling cutting across ethnicities. Conversely, an FGI study conducted in Switzerland revealed that self-sampling offered an opportunity for women to become more at ease with their bodies [35].

The observed link between motherhood and interest in CCU screening, found in our study, is corroborated by the Swiss study [35] that explored the views and acceptability of HPV self-sampling among Swiss women and ethnic minority women. The Swiss study found that a woman’s stage in life had an impact on her willingness to participate in CCU screening. Here, motherhood was considered a facilitator for participation in screening, as this phase reinforced the woman’s sense of responsibility for herself and for others. In particular, it was found that marriage and pregnancy represented a woman’s first opportunity for participation in CCU screening, especially among ethnic minorities [35]. Earlier studies among Muslim women have indicated that a vaginal brush device might enable sexually active yet unmarried younger women to participate in CCU screening without their family’s knowledge [38, 39]. In our study, participants emphasised that non-invasive self-sampling options were the only option for pre-marriage participation in screening. However, our research did not explore perspectives from younger unmarried sexually active women.

The study consistently found that fear of making mistakes during vaginal brush insertion was a prevalent concern. This fear aligns with prior literature involving women of different ethnic backgrounds, including Caucasian women [37, 40–42]. Earlier studies have linked low self-efficacy with reduced likelihood of self-sampling engagement [37, 42], a finding consistent with this study. Addressing low self-efficacy could involve offering clear test instructions and better guidance [38]. A study by Scarinci et al. focused on the acceptability and usefulness of self-sampling for HPV among African American women in the United States [41]. The study found that women’s self-efficacy regarding self-sampling could be fortified through explicit instructions, guidance, and positive experiences. Similar sentiments were expressed among Muslim women in Canada, who indicated that clear instructions for self-sampling tests could bolster low self-efficacy [38].

The women’s positive reception of menstrual blood for screening is encouraging. Despite their aversion to bodily contact with the lower body, they viewed this fluid as natural part of womanhood. Across different cultures, menstruation is often stigmatised and conceptualised as something that is “dirty” or “impure” to be kept private [43, 44]. A Dutch mixed-method study from 2021 investigated, among other groups, ethnic minority women’s attitudes to menstruation in general. Overall, the Dutch population were highly negative towards practices regarding menstruation. Women from non-Dutch ethnicities, had generally more stigmatising attitudes towards menstruation compared to Dutch women [45]. Our study indicates that the negative societal perception of menstrual blood does not necessarily apply to its use within a screening context. This finding suggests the possibility that menstrual blood might find acceptance and recognition within the screening programme.

This study contributes with attitudes and views on four different devices and therefore adds to the literature with a strong insight into preferences for screening methods in ethnic minority populations. The study has provided new knowledge regarding a specially designed menstrual blood device and a first-void urine sample indicating that non-invasive self-sampling may have significant potential for addressing barriers faced by women with non-Western backgrounds. Therefore, it is crucial to consider non-invasive self-sampling alternatives.

### Methodological Considerations

#### Strengths

A main strength of this study lies in its foundation on FGI, which effectively captured nuances in the participants’ attitudes and views on self-sampling [26, 27]. The participants maintained a high level of respect for one another, even when opinions differed, and openly discussed intimate experiences and psychological barriers they had encountered in relation to screening.

#### Limitations

The study could have been improved by providing women with the four different devices in advance for at-home trial before the FGI. This approach would have allowed participants to gain genuine hands-on experience with the methods. Currently, the study gathered immediate views and attitudes on the various methods reflecting what the women might have thought about the devices if they had received them by mail. Future research in this area should consider having participants receive and test the methods beforehand to yield more relevant insights.

On occasion, the participants engaged in brief conversations in their mother tongue. This typically happened when someone encountered language challenges, seeking specific Danish words or assistance in refining their viewpoints. The researchers proactively addressed these instances, seeking to prevent the loss of crucial statements. Despite these efforts, a few conversations were not transcribed.

A challenge that emerged was the non-representative age distribution of the participants in relation to the population invited to participate in the screening programme. Notably, the study did not succeed in recruiting the youngest and oldest women with the participants’ age ranging from 32 to 53. However, it is possible that when discussing younger women’s views and attitudes, participants drew from their own earlier life experiences or reflected on their daughters’ situations, which could indirectly offer a form of representation for younger women.

Based on these methodological considerations the results of the study could hold relevance beyond our research, potentially benefitting ethnic minority women in underserved areas within a publicly funded healthcare system.

### Implications for Practice

The women showed significant interest, curiosity, and preference towards self-sampling via urine and menstrual pad methods. This suggests that non-invasive self-sampling techniques hold promise as effective future strategies to increase participation among non-Western ethnic minority women and simultaneously lower the social inequality in screening. However, it is crucial to conduct additional research to thoroughly confirm the sensitivity and specificity of the samples before considering their inclusion in the screening programme. Furthermore, it is essential to conduct research where women are given the opportunity to use the devices, as this will enhance the validity of the results.

The women were selected with the assumption that non-Western women residing in deprived area in Denmark would exhibit similarities to the general population in Denmark of women with non-Western backgrounds. Despite the overrepresentation of Somali women and elderly women, the study findings may hold relevance for women with non-Western backgrounds in other parts of Denmark, as well as in other countries with similar compositions of ethnic minorities and comparable health systems with free-of-charge services. This perspective is supported by the understanding that shared characteristics such as minority background and healthcare systems could lead to common health behaviours and responses [46], making the study findings potentially relevant to similar Western countries.

## Conclusion

The self-sampling methods, in particular the non-invasive devices, hold significant promise in overcoming some of the barriers towards CCU screening faced by ethnic minority women, positioning it as an essential strategy for the future. The invasive vaginal devices also had potential if the right introduction and guidance were included.

## Data Availability

The datasets used and/or analysed during the current study are available from the corresponding author on reasonable request.

## Abbreviations

*CCU*: Cervical cancer uteri
*GP*: General Practitioner
*HPV*: Human Papillomavirus
*FGI*: Focus group interview(s)
